# Antibody-Mediated Serum Resistance Protects *Pseudomonas aeruginosa* During Bloodstream Infections

**DOI:** 10.1093/infdis/jiad457

**Published:** 2024-01-17

**Authors:** Sarah M Hickson, Johannes K Hoehensteiger, Johanna Mayer-Coverdale, Von Vergel L Torres, Wenkang Feng, Joshua N Monteith, Ian R Henderson, Kate L McCarthy, Timothy J Wells

**Affiliations:** Frazer Institute, Faculty of Medicine, The University of Queensland, Brisbane, Australia; Frazer Institute, Faculty of Medicine, The University of Queensland, Brisbane, Australia; UQ Centre for Clinical Research, The University of Queensland, Herston, Australia; Department of Microbiology, Pathology Queensland, Brisbane, Australia; Frazer Institute, Faculty of Medicine, The University of Queensland, Brisbane, Australia; Frazer Institute, Faculty of Medicine, The University of Queensland, Brisbane, Australia; Frazer Institute, Faculty of Medicine, The University of Queensland, Brisbane, Australia; Institute of Molecular Biosciences, The University of Queensland, Brisbane, Australia; Department of Microbiology, Pathology Queensland, Brisbane, Australia; Infectious Diseases Unit, Royal Brisbane and Women's Hospital, Brisbane, Australia; Frazer Institute, Faculty of Medicine, The University of Queensland, Brisbane, Australia; Australian Infectious Diseases Research Centre, The University of Queensland, Brisbane, Australia

**Keywords:** bloodstream infections, host-pathogen interactions, protective antibodies, *Pseudomonas aeruginosa*, serum resistance

## Abstract

**Background:**

*Pseudomonas aeruginosa* is a frequent pathogen isolated from bacterial bloodstream infection (BSI) and is associated with high mortality. To survive in the blood, *P aeruginosa* must resist the bactericidal action of complement (ie, serum killing). Antibodies usually promote serum killing through the classical complement pathway; however, “cloaking antibodies” (cAbs) have been described, which paradoxically protect bacteria from serum killing. The relevance of cAbs in *P aeruginosa* BSI is unknown.

**Methods:**

Serum and *P aeruginosa* were collected from a cohort of 100 patients with BSI. Isolates were tested for sensitivity to healthy control serum (HCS). cAb prevalence was determined in sera. Patient sera were mixed with HCS to determine if killing of the matched isolate was inhibited.

**Results:**

Overall, 36 patients had elevated titers of cAbs, and 34 isolates were sensitive to HCS killing. Fifteen patients had cAbs and HCS-sensitive isolates; of these patients, 14 had serum that protected their matched bacteria from HCS killing. Patients with cAbs were less likely to be neutropenic or have comorbidities.

**Conclusions:**

cAbs are prevalent in patients with *P aeruginosa* BSI and allow survival of otherwise serum-sensitive bacteria in the bloodstream. Generation of cAbs may be a risk factor for the development of BSI.

## Teaser

Cloaking antibodies (cAbs) paradoxically protect bacteria from serum killing. Here we show that cAbs are prevalent in patients with *Pseudomonas aeruginosa* BSIs and allow survival of otherwise serum-sensitive bacteria. Generation of cAbs may increase susceptibility to bloodstream infections.

*Pseudomonas aeruginosa* bloodstream infections (BSIs) remain a major cause of morbidity and mortality worldwide. *P aeruginosa* is 1 of the top 5 causes of BSI overall and is even more common in settings such as intensive care units [1, 2]. *P aeruginosa* BSI is associated with a higher mortality rate than other bacterial BSIs, with mortality between 27% and 48% [2–4]. BSIs are also associated with excess long-term mortality in patients [5, 6]. In particular, *P aeruginosa* is associated with a high incidence of mortality due to its increasing resistance to ceftazidime and imipenem [1, 7–9]. Indeed, *P aeruginosa* is 1 of only 3 species of bacteria listed by the World Health Organization as a critical priority for new antibiotic development due to the rise in antimicrobial resistance [10].

BSIs often originate from a primary source infection, where bacteria cross epithelial and endothelial barriers to enter the bloodstream [11, 12]. Common sources of *P aeruginosa* BSI include respiratory, urinary tract, and line-related infections (eg, central venous catheter) [7, 8, 13, 14]. Bacterial survival in the bloodstream is crucial for the development of a BSI, a difficult proposition for gram-negative bacteria due to their outer membrane being susceptible to serum killing by the complement system.

The complement system consists of many plasma proteins that, when triggered, cascade to induce a series of inflammatory responses. It can also lead to rapid bactericidal killing by the formation of the membrane attack complex (MAC), which forms a pore in the outer membrane of gram-negative bacteria such as *P aeruginosa* [15]. This MAC-dependent killing is commonly known as *serum killing*. Gram-positive bacteria are intrinsically resistant to serum killing due to the MAC not being able to penetrate the thick peptidoglycan layer [16]. In contrast, gram-negative bacteria such as *P aeruginosa* must produce factors to resist serum killing, including the O-antigen component of lipopolysaccharide (LPS) [17–20]. Despite this, a significant proportion (27%) of bloodstream isolates are still sensitive to the action of healthy control serum (HCS) [21].

Antibodies usually promote serum killing of *P aeruginosa* through the classical complement-mediated pathway. However, paradoxically, some specific antibody has been found to shield bacteria from serum killing, thus enabling the continued infection of the host. These “cloaking antibodies” (cAbs) are high titers of IgG2 or IgA specific for the O-antigen component of LPS and are hypothesized to inhibit complement killing by creating a physical barrier distal from the bacterial surface preventing MAC insertion into the membrane [22–24]. cAbs have been described against a range of gram-negative bacterial species, including *Salmonella enterica*, *Escherichia coli*, and *Proteus mirabilis* [24–27]. These antibodies are also prevalent (20%–40%) in the blood of patients with various *P aeruginosa* respiratory infections, where the presence of cAbs correlates with worse lung function and outcomes [22, 23, 28]. Despite these findings, the field does not currently know the prevalence and impact of cAbs in patients with *P aeruginosa* BSI, where serum resistance is vital for bacterial survival. Here we investigate 100 patients with *P aeruginosa* BSI and determine the clinical relevance of cAbs.

## METHODS

### Patient Samples and Isolates

Serum samples and matched bacterial isolates were collected from 100 patients with *P aeruginosa* BSI, identified by the routine laboratory from 3 hospitals in Brisbane, Australia: Royal Brisbane Hospital, Princess Alexandra Hospital, and Prince Charles Hospital. Deidentified data, including age, sex, comorbidities, source of infection, antibiotic treatment, and disease outcome, were collected from patients' medical health records.

*P aeruginosa* were isolated from patient blood and grown under aerobic conditions on lysogeny broth agar plates (37 °C). Single colonies were cultured in suspension in lysogeny broth medium (37 °C, with shaking at 200 rpm). All patient sera were complement inactivated at 56 °C for 20 minutes. To remove the effects of any antibiotics in patients’ blood, for some of the experiment, serum was buffer exchanged (5-kDa Vivaspin columns) into an equal volume of phosphate-buffered saline (PBS) retaining similar titers of antibody (Supplementary Figure 1). *P aeruginosa* isolates were serotyped via polymerase chain reaction with specific primers, as previously described [29, 30] (Supplementary Table 1).

### LPS Extraction and Visualization

LPS was extracted in 2 methods depending on the purity required. To examine O-antigen expression of clinical isolates, the “quick” proteinase K extraction method was used, as previously described [23]. The LPS used for enzyme-linked immunosorbent assay (ELISA) screening was purified from a panel of representative O-antigen–type strains via the hot phenol–water method, as described by Wright and Rebers in 1972 [31]. LPS extracts were separated via electrophoresis on 4%–12% Bis/Tris gels (Life Sciences) and visualized through 2 methods: staining with the Pierce Silver Stain Kit (Invitrogen) or Western blotting. For blotting, LPS was transferred to polyvinylidene difluoride membranes with the iBlot 2 Gel Transfer Device (Invitrogen) as recommended. The membranes were blocked in Blotto (5% nonfat milk, 0.05% Tween 20, and PBS [pH 7.4]) before incubation in patient serum (1:10 000 in Blotto). A secondary antibody—alkaline phosphatase (AP)–conjugated anti-human IgG2 or IgA diluted 1:10 000 in Blotto—was used to detect bound patient antibody. Membranes were developed with nitro-blue tetrazolium and 5-bromo-4-chloro-3′-indolyphosphate (Thermo Fisher). Both these methods were used to verify extraction of the 9 LPS serotypes used for screening (Supplementary Figure 2). LPS was quantified by comparison to standard dilutions of commercially available *P aeruginosa* LPS (Sigma-Aldrich).

### Enzyme-Linked Immunosorbent Assay

Antibody titer in patient sera was calculated via an ELISA toward the matched LPS serotype with a panel of 9 O-antigens [26]. HCS was pooled from at least 6 healthy donors and used as a negative control in ELISAs. Briefly, plates were coated with 1 µg/mL of the appropriate O-antigen. Three patient isolates were O9 or O13/14 serotypes. As these serotypes are not covered in our panel, we could not determine matched antibody titer in these 3 patients' sera. Patient serum was added as either a single dilution (1:500) or a 3-fold dilution (1:20–1:4860) before washing and addition of secondary antibody (anti-human IgG-AP, anti-human Ig [polyvalent]–AP, anti-human IgG2 [Fc specific], anti-human IgA, 1:2000; Sigma) and, where needed, a tertiary antibody (anti-mouse IgG-AP, 1:2000; Sigma). Absorbance readings were determined after 15 and 30 minutes, as detected with p-nitrophenyl phosphate substrate solution at 405 nm.

### Serum Bactericidal Assay

Killing of *P aeruginosa* isolates in serum was determined via serum bactericidal assay as previously described [22]. Briefly, buffer-exchanged patient sera were complement inactivated at 56 °C for 30 minutes. Cultures were grown to a stationary phase: 1.5 mL of overnight culture was adjusted to an OD_600_ of 0.6 (optical density at 600 nm), pelleted via centrifugation, and resuspended in 1 mL of PBS, creating a stock inoculum. A 50:50 mix of HCS (active complement) and patient sera (inactive complement/antibody source) or 1× PBS (positive control) was added to create a 25-μL reaction with 2.5-μL 1:10 diluted stock in 1× PBS; 45 μL of 1× PBS with diluted stock (1:10) was used as a negative control. Reactions were incubated at 37 °C with 5 μL removed at 45, 90, and 180 minutes. The removed aliquot was serially diluted 10-fold, plated to lysogeny broth agar, and incubated overnight at 37 °C. Colony-forming units per milliliter were determined to calculate fold change in bacterial growth as compared with the stock inoculum’s growth. The area under the curve was compared to determine significance in growth fold change. Exponentially grown cultures (OD_600_, 0.6) for 12 strains were tested in a serum bactericidal assay with HCS:PBS, and despite a trend of higher killing of exponential-phase strains, no significant difference was found between cultures grown in the exponential and stationary phases (Supplementary Figure 3).

### Statistics

All data analysis was conducted with Prism software (version 8.0.1; GraphPad). A power calculation was used to determine patient numbers predicting a cAb incidence of 20% based on previous cohorts (21%–41%), giving sufficient power to identify any significance between serum sensitivity and other patient factors [22, 23, 28]. Patient data were analyzed for significance with a Fisher exact test. Serum bactericidal data were analyzed by comparing significant differences of the area under the curve with a Student *t* test. *P* < .05 was considered significant.

### Ethical Approval

This study was approved by the Human Research Ethics Committee of Royal Brisbane and Women’s Hospital (HREC/2018/QRBW/49202). HCS was collected with ethics approval of the Metro South Human Research Ethics (HREC/2019/QMS/54445). The research project meets the requirements of the National Health and Medical Research Council's National Statement on Ethical Conduct in Human Research (2007).

## RESULTS

### Patient Characteristics

One hundred patients with *P aeruginosa* BSI were recruited (Table 1, Supplementary Table 2). Across the 100 patients, over half were male (67%), and ages ranged between 9 and 92 years, with the majority being >50 years old (79%). Most infections were acquired in a health care setting (70%) by patients with comorbidities (75%), as indicated by a Charlson Comorbidity Index (CCI) >0. Sources of infection were classified into broad categories, with urinary and respiratory tracts as frequent sources. Despite antibiotic treatment, 9 of the 100 patients died within 30 days of presentation, with no correlation found between patient death and source of infection.

**Table 1.** Patient Factors Associated With Lipopolysaccharide-Specific Antibodies or cAbs in Serum

Patient Factor	Total (N = 100)	cAb (n = 36)	No cAb (n = 64)	*P* Value
Source of infection				
Gastrointestinal	8	3	5	>.9999
Skin	11	6	5	.1964
Line related	7	3	4	.6999
Urinary	22	11	11	.1376
Respiratory	10	6	4	.1615
Neutropenic	39	7	32	.0029***
CCI				
0	24	15	9	.0031***
1 or 2	46	12	34	.2806
3 or 4	17	4	13	.2806
≥5	13	5	8	>.9999
Death at 30 d	9	3	6	>.9999
Sex				.5077
Male	67	26	41	
Female	33	10	23	
Acquisition				.0705
CAI	30	15	15	
HAI/HCAI	70	21	49	
Previous *P aeruginosa* infection	12	4	8	>.9999

Abbreviations: cAb, cloaking antibody; CAI, community-acquired infection; CCI, Charlson Comorbidity Index; HAI, hospital-acquired infection; HCAI, healthcare–associated infection; *P aeruginosa*, *Pseudomonas aeruginosa*.

***P < .001.

### Thirty-four Percent of *P aeruginosa* Blood Isolates Are Sensitive to Serum-Mediated Killing

**Figure 1.**
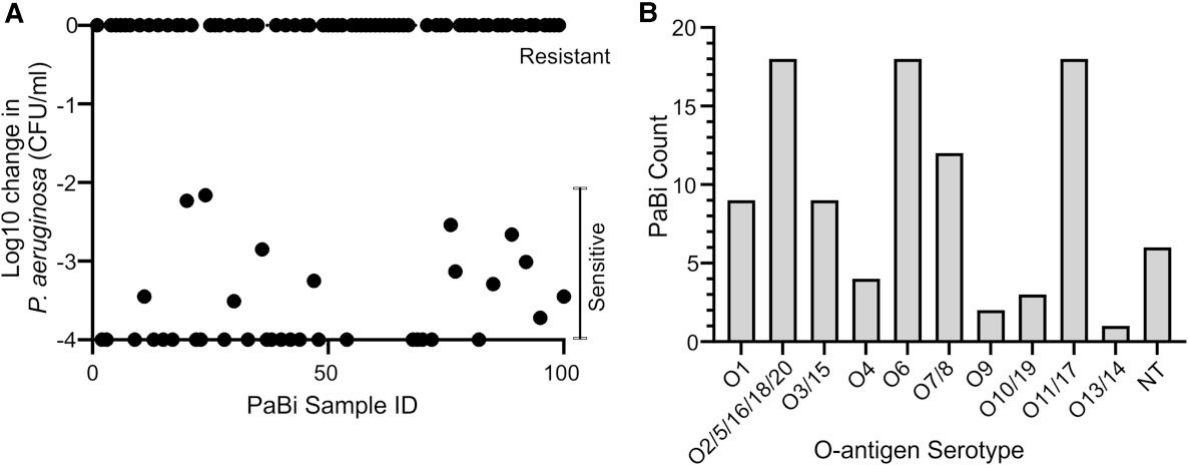
Patient isolate susceptibility to HCS killing. *A*, Patient isolate (PaBi) survival in 50:50 HCS:PBS and PBS alone was compared to determine *Pseudomonas aeruginosa* sensitivity to serum killing. Difference in bactericidal activity of HCS was determined by measuring CFU per milliliter after 3-hour incubation, with significance reported via Student *t* test, *P* > .05 (n = 3). Isolates that have no statistically significant difference vs PBS alone are placed at 0 on the graph. *B*, Distribution of PaBi serotypes. Due to similarities of genetic regions, some serotype groups are clustered. CFU, colony-forming units; HCS, healthy control serum; NT, nontypable; PBS, phosphate-buffered saline.

Matched serum and bacterial isolates from the blood were collected from all patients. We first determined if the isolates were resistant to HCS killing. All isolates were incubated in HCS, and survival was measured after 3 hours. Despite all strains being isolated from blood, 34 of 100 (34%) were sensitive to HCS killing (<1% survival; Figure 1*A*). We examined the LPS phenotype of these 34 strains, as O-antigen expression is a major determinant of serum resistance. Of these 34 strains, 27 (79%) had detectable O-antigen expression despite being sensitive to HCS killing (Supplementary Figure 4). Interestingly, all O4 (3/3) and O10 (4/4) isolates were sensitive to HCS while maintaining O-antigen expression (Supplementary Table 2). As cAbs bind to O-antigen, it may be that these 27 strains are surviving in patient blood through antibody-mediated serum resistance.

### Thirty-six Percent of Patient Sera Have High Relative Titers of LPS-Specific Antibodies

**Figure 2.**
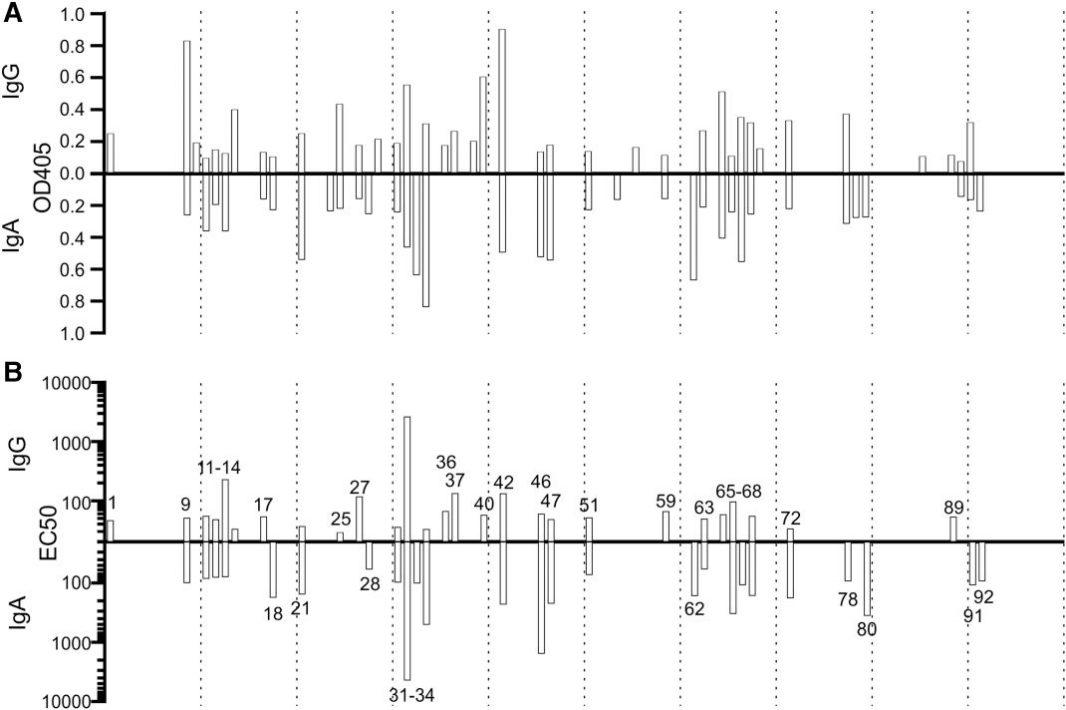
Sera antibody responses to *Pseudomonas aeruginosa* LPS. *A*, LPS-specific IgG and IgA response of patient serum was measured against the cognate strains matched serotype of LPS. A high response of antibody to LPS was defined as an OD_405_ reading 3 times the SEM of the HCS response to serotyped LPS (n = 44). *B*, The titer of LPS-specific IgG and IgA in patient serum was calculated against the cognate strain serotype of LPS. A significant titer of cloaking antibody was defined as an EC_50_ value greater than the HCS to serotyped LPS (n = 36). EC_50_, half maximal effective concentration; HCS, healthy control serum; LPS, lipopolysaccharide; OD_405_, optical density at 405 nm.

Antibody-mediated serum resistance is facilitated by cAbs that are typically specific to the O-antigen component of LPS [22]. Genetic serotyping of the isolates revealed a wide representation of isolates across all serotype groups, except O12, and found that no single serotype dominated the cohort (Figure 1*B*). LPS-specific antibodies of IgG, IgA, and IgM isotypes were measured against a panel of purified LPS that matched the cognate strain serotype. Of the 100 patient sera, 45 generated responses of either IgG or IgA to LPS of their cognate serotype significantly above that of HCS (Figure 2*A*). LPS-specific IgM responses were low across the cohort and thus not further investigated (data not shown). The titer EC_50_ (half maximal effective concentration) of LPS-specific antibody of the sera with high initial responses was measured by ELISA. Of the samples investigated (n = 45), 36 patient sera had an EC_50_ >50, indicative of the presence of cAbs in our previous study (Figure 2*B*) [28]. Patients with O2 (11/18) and O3 (7/9) serotype isolates were significantly more likely to have cAbs in their sera (Supplementary Table 2). Thus, 36% of patients with *P aeruginosa* BSI have high-enough titers of cAbs likely to inhibit serum killing of the bacteria.

### Patient Sera Inhibit Serum-Mediated Killing of Cognate Isolates

**Figure 3.**
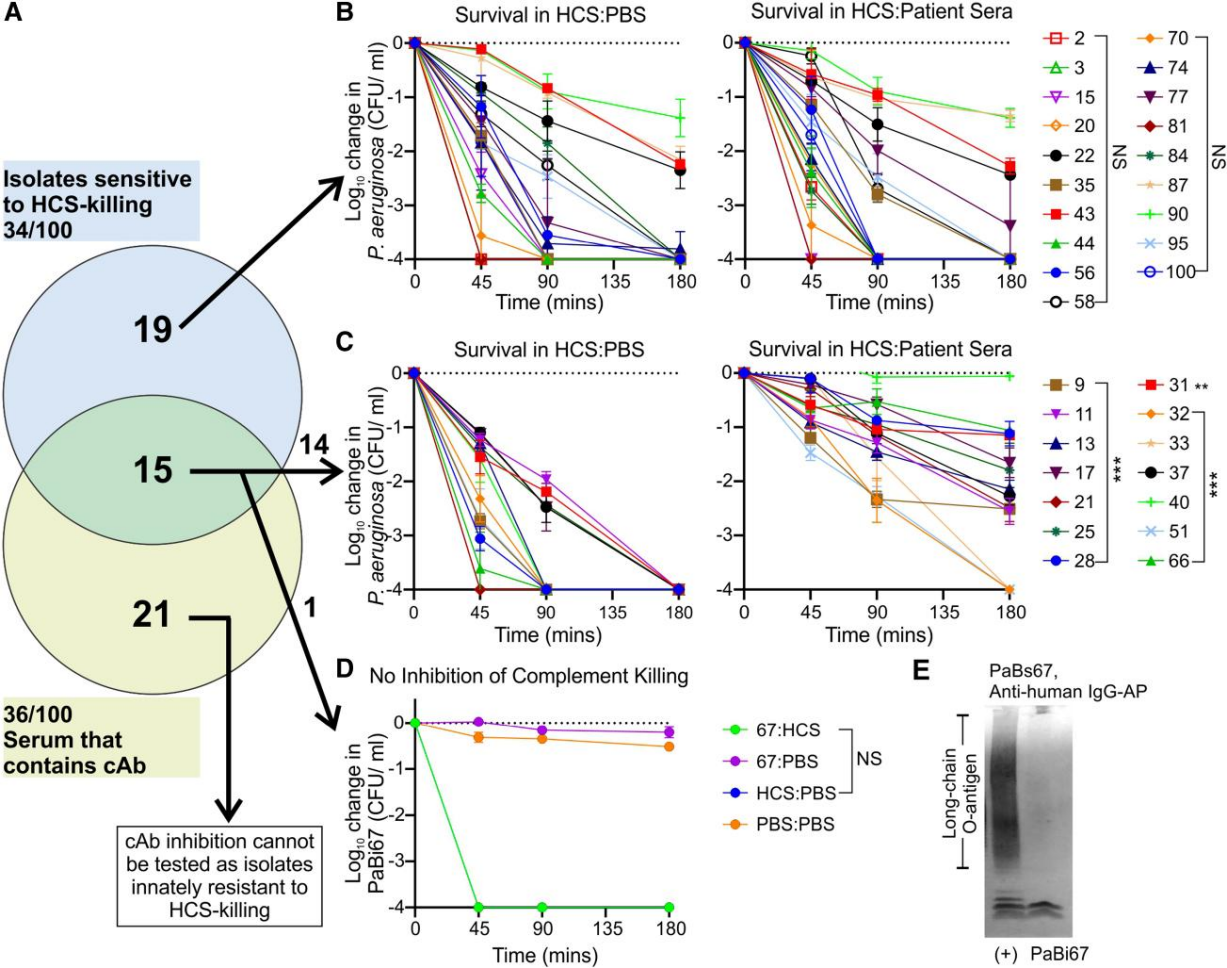
Inhibition of HCS killing. *A*, Relationship between isolates that are sensitive to HCS killing and patient serum that contains cAbs. *B*–*D*, Serum bactericidal killing of each patient’s cognate *Pseudomonas aeruginosa* isolate (PaBi) incubated with either 50:50 mix of HCS:PBS or 50:50 mix of HCS:matched patient serum (PaBs). *B*, Isolates sensitive to HCS killing with cAb-negative matched serum (n = 19) had no significant difference in killing when incubated in HCS mixed 50:50 with PBS or matched patient serum. *C*, Of 15 HCS-sensitive isolates with cAb-positive matched serum, 14 had significantly less bactericidal killing when patient sera were mixed 50:50 with HCS. *D*, PaBi67 did not have significant inhibition of HCS killing after addition of matched patient serum despite presence of cAbs. *E*, Western blotting of PaBi67 and O-antigen–positive LPS with PaBi67 sera reveals that PaBi67 does not produce long-chain O-antigen. Significant inhibition of HCS killing was determined via Student *t* test. **P < .01. ***P < .001. Error bars represent SD; n = 3 for all sera. cAb, cloaking antibody; CFU, colony-forming units; HCS, healthy control serum; LPS, lipopolysaccharide; NS, not significant; PBS, phosphate-buffered saline.

The presence of cAbs in serum is relevant to bacterial survival in blood only if the matched patient isolate is innately sensitive to the action of HCS. Of the 34 strains that were HCS sensitive, 15 were isolated from patients with sera containing cAbs (Figure 3*A*). To verify that the presence of these antibodies inhibited the action of HCS complement, serum-killing assays were performed with cognate patient serum of the 34 serum-sensitive isolates mixed 1:1 with HCS and compared with HCS alone. As expected, the 19 sera without cAbs were unable to inhibit the bactericidal action of HCS (Figure 3*B*, Supplementary Figure 5). In contrast, 14 of 15 patient sera that contained cAbs significantly inhibited the bactericidal action of HCS (Figure 3*C*, Supplementary Figure 6). Serum/isolate PaBi67 was the sole exception, with no inhibition detected despite cAbs present in PaBs67 (Figure 3*D*). PaBi67 is O-antigen negative (Figure 3*E*), thus explaining the lack of inhibition despite the presence of cAbs. Overall, serum-killing inhibition of patient isolates with sera was observed in 14 of 100 patients and almost half the innately serum-sensitive isolates (14/34). Thus, antibody-mediated serum resistance via cAbs may have allowed these strains to survive in the bloodstream and cause infection.

### Clinical Associations With cAbs

All patient data—including age, CCI risk factors, treatment, outcome, sources of infection, and previous *P aeruginosa* infection—were statistically compared between patients with cAbs (n = 36) and without (n = 64; Table 1). Patients with neutropenia and those with no comorbidities (CCI, 0) were significantly more likely to lack cAbs. No statistical differences were found in outcome, age, treatment, or source of BSI. When we focused on the 14% of patients who had relevant cAbs, only nonneutropenia was associated with presence of cAbs in the cohort (Table 2). Thus, while cAbs may allow the development of BSI for patients with otherwise serum-sensitive bacteria, the presence of these antibodies, once BSI develops, does not seem to affect patient outcome.

**Table 2.** Patient Factors Associated With Inhibition of HCS Killing via Antibodies in Serum

Patient Factor	Inhibition of HCS Killing (n = 14)	No Inhibition (n = 86)	*P* Value
Source of infection			
Gastrointestinal	1	6	.0802
Skin	2	9	>.9999
Line related	2	5	.2613
Other	0	8	.5970
Urinary	5	17	.2960
Respiratory	1	9	>.9999
Neutropenic	2	37	.0414*
CCI			
0	5	19	.3249
1 or 2	7	38	.5609
3 or 4	1	17	.4529
≥5	1	12	.6849
Death at 30 d	2	7	.6136
Sex			.3716
Male	11	54	
Female	3	30	
Acquisition			.6546
CAI	5	25	
HAI/HCAI	9	59	

Abbreviations: CAI, community-acquired infection; CCI, Charlson Comorbidity Index; HAI, hospital-acquired infection; HCAI, healthcare–associated infection; HCS, healthy control serum.

^*^ *P* < .05.

## DISCUSSION

*P aeruginosa* BSIs have been extensively investigated due to the high morbidity and mortality rates, commonly focusing on the efficacy of antimicrobial treatment [13, 32]. However, to first cause BSI, the bacterium must survive in the blood. Here we find that 34% of *P aeruginosa* isolated from the blood was sensitive to the serum killing of HCS. To determine if antibody-mediated serum resistance via protective cAbs could be protecting *P aeruginosa* in the blood, we investigated the prevalence of cAbs in this cohort. Of the 100 patient isolates in our cohort, 36% had high-enough titers of cAbs in the blood to protect *P aeruginosa* from serum killing. We also demonstrated in 14 patients that these cAbs from their sera could protect their matched isolates from HCS killing. Thus, we have shown that the cAb mechanism is relevant in acute BSI and can enable the survival of bacterial strains in the blood that would otherwise have been cleared by complement.

Serum resistance is a key virulence trait of gram-negative bacteria causing BSIs, as complement killing can otherwise kill sensitive bacteria within minutes of activation [15]. To overcome this, bacteria have a plethora of virulence factors specific to evading host complement [33, 34]. Surprisingly, given that all *P aeruginosa* was isolated from the bloodstream, 34% of our cohort was sensitive to complement-mediated killing by HCS. Previous studies reported serum sensitivity in 27% of gram-negative BSI isolates, while others suggested a rapid increase of serum resistance throughout infection [21, 35]. Some serum-sensitive strains may be able to survive in blood due to patients being immune compromised [8]. Here we found that 44% of serum-sensitive isolates were from patients with cAbs, suggesting that these antibodies are a relevant mechanism enabling bacterial survival in the blood in *P aeruginosa* acute infections. It is currently unknown how the other 19 serum-sensitive isolates whose patients did not have cAbs were surviving in the blood.

Despite the acute nature of BSI, we found that 36% of patients produced high titers of IgG and IgA to their cognate bacterial LPS. Typically, IgM is the first antibody to respond in bacterial infections, with IgG and IgA developing over the first 2 weeks [36]. Only 12 of the 100 patients had a record of previous *P aeruginosa* infection, suggesting that the LPS-specific IgG and IgA may have developed during the current hospitalization. The majority of *P aeruginosa* BSIs are hospital acquired, often associated with line-related infections, such as catheter-associated urinary tract infections and ventilator-associated pneumonia, supplying an indwelling device with a constant source of bacteria and promoting antibody development [37, 38]. Studies show that the likelihood of acquiring *P aeruginosa* increases by 1.4% per week of hospitalization, with a 23% chance by the 10th week of admission [39]. Length of stay in the hospital has been shown as an independent risk factor for the development of a BSI. Thus, many patients with *P aeruginosa* BSI have likely been in the hospital for a sufficient time to generate an IgG or IgA response. To determine if the generation of cAbs increases susceptibility to *P aeruginosa* BSI, longitudinal studies measuring cAb titers from admission will need to be carried out.

In contrast, neutropenia has been established as a risk factor for BSI, and reported time to positive culture was <24 hours in patients with neutropenia and *P aeruginosa* BSI [40, 41]. According to recorded hospitalization data, patients with neutropenia or a CCI of 0 were less likely to have cAbs. The lack of association with neutropenia is likely due to a lack of antigen presentation by neutrophils to the adaptive immune system. It may also be that patients did not have sufficient time to develop antibodies against their cognate *P aeruginosa* isolates in these cases.

A limitation of this study is that by using serum, we cannot determine the other effector functions of O-antigen–specific IgG2 or IgA, such as opsonization and phagocytosis relevant to BSIs. However, IgG2 and IgA have poor recognition by Fc receptors, which may make them poor opsonins [42]. Indeed, IgG2 antibodies have been seen to exert antiphagocytic effects on *P aeruginosa* [43], and LPS-specific antibodies have been shown to inhibit uptake of the bacteria in alveolar macrophages [44–46]. Future studies should focus on whether these mechanisms are also relevant for cAbs in BSIs.

cAbs exist against a range of bacterial infections, including *E coli*, *Salmonella*, *Proteus*, *Klebsiella*, *Burkholderia*, *Neisseria* sp, and *Brucella* [24, 25]. We previously demonstrated that 24% of patients with *E coli* urosepsis possess cAbs and that strains isolated from patients with cAbs are more sensitive to HCS killing [26]. For these reasons, the serum resistance mechanism of cAbs could similarly protect multiple other gram-negative bacterial species frequently causing BSI.

Importantly, we showed in previous studies that removing cAbs can restore serum killing of *P aeruginosa*, regardless of multidrug resistance [22, 28]. Indeed, 3 patients with multidrug-resistant *P aeruginosa* respiratory infections and cAbs have received plasmapheresis to remove cAbs and restore serum killing of the strains [23, 28]. In all cases, after plasmapheresis, patients had no detectable *P aeruginosa* and were able to return home. Plasmapheresis is a broad treatment removing all protein from the blood, including cytokines and protective antibody, and is not a viable treatment option for patients with rapidly progressing infections such as BSI. However, methods that could directly target and remove cAbs or interfere with their binding have the potential to restore the serum killing of multidrug-resistant isolates.

## Supplementary Data

Supplementary materials are available at *The Journal of Infectious Diseases* online (http://jid.oxfordjournals.org/). Supplementary materials consist of data provided by the author that are published to benefit the reader. The posted materials are not copyedited. The contents of all supplementary data are the sole responsibility of the authors. Questions or messages regarding errors should be addressed to the author.

## Supplementary Material

jiad457_Supplementary_Data

## Notes

***Author contributions*.** Designing research studies; S. M. H., J. K. H., K. L. M., T. J. W. Conducting experiments; S. M. H., J. K. H., V. V. L. T., W. F., J. N. M. Acquiring data; J. M.-C., K. L. M. Analyzing data; S. M. H., J. K. H., K. L. M., I. R., T. J. W. Writing the manuscript S. M. H., J. K. H., T. J. W. Editing and reviewing manuscript: all authors.

***Financial support*.** This work was supported by the Study, Education and Research Committee of Pathology Queensland (grant 5905 to K. L. M. and T. J. W.); and a grant from the Australian Government to the Translational Research Institute, where this research was carried out. Funding to pay the Open Access publication charges for this article was provided by the Australia and New Zealand Institutions (CAUL affiliated).
