# Supplementary material for: Antibody-Mediated Serum Resistance Protects Pseudomonas aeruginosa During Bloodstream Infections
Source: J Infect Dis. 2024 Jan 17;230(2):e221–9. doi: 10.1093/infdis/jiad457 (PMC11326846; doi:10.1093/infdis/jiad457)

Supplementary Data

Supplementary Table 1. **Primers used to classify *P. aeruginosa* isolates from patients with bacterial bloodstream infections via PCR.**

| **Serotype** | **Forward (5’-3’)** | **Reverse (5’-3’)** |
| --- | --- | --- |
| **O1** | TGCTCTTTGGCTTCTTGATTCTTG | TCTACACCGCCAGAACTACCTAGCT |
| **O2/5/16/18/20** | GCGGCTTTTATCAACCGTGTCGCA | TCCCTTGGCACACTGGAAGGACAT |
| **O3/15** | GCGTCGTTGTTCAGTTTGGACGTG | TCTGGAAAACCTGAGCAGCCGTCC |
| **O4** | GCTAATAACGGAAGGACCTTGAAT | TAAAAGCTCGGCGTAACGCTTATG |
| **O6** | ATTGGCTAGTGCTACACGAGTGCA | CGAATTAGCTTGCTCTTCAGGAAAG |
| **O7/8** | GGAATGTCGCTCTCGTTTCAAGTG | CTAGTATTCATCAACTGCTGTAC |
| **O9** | GGAACATTGGAATCAAGAGGTTATG | CCACTAAATACCAGGCATACAACTA |
| **O10/19** | CAGCAGGGAATATCGCTTGAACAGT | CTATAATGCATTAGCGACTCACCG |
| **O11/17** | TACTTCACCCATAGCTAGCGCTCTA | TTCTCTCTCAACTTAACCGTGGCC |
| **O12*** | ATGAAAAAAGTTTTGGTTACTGGG | CCCTCTCGAATCGAGTAGGTAGGCTC |
| **O13/14** | ACTATTCAGTCGATGATCCTTGTG | AGCAGTTAGCAAATTTCACTCTCCAGCC |

Supplementary Table 2. Cohort characteristics

| PaB ID | LPS Serotype | HCS-Sensitive Isolate | cAbs | Inhibits | Patient Data | | | | | |
| --- | --- | --- | --- | --- | --- | --- | --- | --- | --- | --- |
|  |  |  |  |  | **Previous Infection** | **CCI** | **Np** | **Infection Source Classification** | **Death at 7 days** | **Death at 30 days** |
| 1 | O2/5/16/ 18/20 |  | X |  | N | 3 | Y | GI | N | N |
| 2 | O9 | X |  |  | N | 2 | N | Urinary | N | N |
| 3 | O3/15 | X |  |  | Y | 3 | N | Other | N | N |
| 4 | O6 |  |  |  | N | 3 | Y | GI | N | N |
| 5 | O6 |  |  |  | N | 0 | N | Skin | N | N |
| 6 | O2/5/16/ 18/20 |  |  |  | N | 5 | Y | GI | Y |  |
| 7 | O3/15 |  |  |  | N | 2 | N | Unknown | N | N |
| 8 | O7/8 |  |  |  | N | 2 | Y | GI | N | N |
| 9 | O7/8 | X | X | X | N | 8 | Y | Skin | N | N |
| 10 | O7/8 |  |  |  | Y | 6 | N | GI | N | N |
| 11 | O10/19 | X | X | X | N | 1 | N | Respiratory | N | N |
| 12 | O2/5/16/ 18/20 |  | X |  | N | 3 | N | Urinary | N | N |
| 13 | O1 | X | X | X | N | 0 | N | Urine | N | N |
| 14 | O2/5/16/ 18/20 |  | X |  | Y | 0 | N | GI | N | N |
| 15 | O10/19 | X |  |  | N | 3 | N | Urinary | N | N |
| 16 | O2/5/16/ 18/20 |  |  |  | N | 4 | Y | GI | N | N |
| 17 | O4 | X | X | X | N | 2 | Y | GI | N | N |
| 18 | O6 |  | X |  | N | 0 | N | Urinary | N | N |
| 19 | O6 |  |  |  | N | 2 | Y | Line | N | N |
| 20 | O6 | X |  |  | N | 1 | N | Other | N | N |
| 21 | O2/5/16/ 18/20 | X | X | X | N | 0 | N | Urinary | N | N |
| 22 | O1 | X |  |  | N | 0 | N | Urinary | N | N |
| 23 | O11/17 |  |  |  | Y | 2 | Y | Other | N | N |
| 24 | O6 |  |  |  | N | 2 | Y | GI | N | N |
| 25 | O6 | X | X | X | N | 0 | N | Unknown | N | N |
| 26 | O11/17 |  |  |  | N | 2 | Y | Line | N | N |
| 27 | O2/5/16/ 18/20 |  | X |  | N | 1 | N | Unknown | N | Y |
| 28 | O6 | X | X | X | N | 2 | N | Line | N | Y |
| 29 | O7/8 |  |  |  | N | 2 | Y | Skin | N | N |
| 30 | O2/5/16/ 18/20 |  |  |  | N | 6 | N | Unknown | N | N |
| 31 | O6 | X | X | X | N | 2 | N | Urinary | N | N |
| 32 | O3/15 | X | X | X | N | 3 | N | Urinary | N | N |
| 33 | O2/5/16/ 18/20 | X | X | X | Y | 2 | N | Line | N | N |
| 34 | O2/5/16/ 18/20 |  | X |  | N | 0 | N | Other | N | N |
| 35 | O4 | X |  |  | N | 2 | Y | GI | N | N |
| 36 | O11/17 |  | X |  | N | 5 | N | Skin | N | N |
| 37 | O4 | X | X | X | N | 0 | N | Unknown | Y |  |
| 38 | O3/15 |  |  |  | N | 0 | N | GI | N | N |
| 39 | O6 |  |  |  | N | 2 | Y | Respiratory | Y |  |
| 40 | O4 | X | X | X | N | 2 | N | GI | N | N |
| 41 | O2/5/16/ 18/20 |  |  |  | N | 2 | N | Skin | N | N |
| 42 | O2/5/16/ 18/20 |  | X |  | N | 0 | N | Respiratory | N | N |
| 43 | O7/8 | X |  |  | N | 2 | Y | GI | N | N |
| 44 | O9 | X |  |  | Y | 5 | N | Respiratory | N | N |
| 45 | O11/17 |  |  |  | N | 2 | Y | GI | N | N |
| 46 | O3/15 |  | X |  | Y | 2 | N | Respiratory | N | N |
| 47 | O11/17 |  | X |  | N | 0 | Y | GI | N | N |
| 48 | O11/17 |  |  |  | N | 2 | Y | GI | N | N |
| 49 | O6 |  |  |  | N | 3 | N | Skin | N | N |
| 50 | O11/17 |  |  |  | N | 2 | Y | GI | N | N |
| 51 | O11/17 | X | X | X | N | 2 | N | Urinary | N | N |
| 52 | O7/8 |  |  |  | N | 1 | N | Respiratory | N | N |
| 53 | O1 |  |  |  | Y | 0 | N | Respiratory | N | N |
| 54 | O2/5/16/ 18/20 |  |  |  | Y | 0 | N | Respiratory | N | N |
| 55 | O6 |  |  |  | N | 6 | N | Respiratory | Y |  |
| 56 | NT | X |  |  | N | 1 | N | Unknown | N | N |
| 57 | O11/17 |  |  |  | N | 2 | Y | GI | N | N |
| 58 | NT | X |  |  | N | 3 | Y | GI | N | N |
| 59 | O11/17 |  | X |  | N | 0 | N | Skin | N | N |
| 60 | O1 |  |  |  | N | 1 | N | Urinary | N | N |
| 61 | O7/8 |  |  |  | N | 2 | Y | GI | N | N |
| 62 | O2/5/16/ 18/20 |  | X |  | N | 0 | N | Respiratory | N | N |
| 63 | O6 |  | X |  | N | 6 | N | Urinary | N | N |
| 64 | O3/15 |  |  |  | N | 3 | N | Urinary | N | N |
| 65 | O2/5/16/ 18/20 |  | X |  | N | 0 | N | Skin | N | N |
| 66 | O1 | X | X | X | N | 0 | N | Skin | N | N |
| 67 | O2/5/16/ 18/20 | X | X |  | N | 0 | N | Other | N | N |
| 68 | O7/8 |  | X |  | N | 2 | Y | Urinary | N | N |
| 69 | O6 |  |  |  | N | 7 | N | Urinary | N | N |
| 70 | O1 | X |  |  | N | 2 | N | Line | N | N |
| 71 | O11/17 |  |  |  | N | 2 | Y | GI | Y |  |
| 72 | O7/8 |  | X |  | N | 3 | N | Unknown | N | N |
| 73 | O1 |  |  |  | N | 2 | Y | GI | N | N |
| 74 | O6 | X |  |  | N | 6 | N | GI | N | N |
| 75 | NT |  |  |  | N | 2 | Y | GI | N | N |
| 76 | NT |  |  |  | N | 6 | N | GI | N | N |
| 77 | O11/17 | X |  |  | N | 0 | N | Urinary | N | N |
| 78 | O7/8 |  | X |  | Y | 6 | Y | Line | N | N |
| 79 | O2/5/16/ 18/20 |  |  |  | N | 3 | N | Urinary | N | N |
| 80 | O6 |  | X |  | N | 2 | N | Other | N | N |
| 81 | O11/17 | X |  |  | Y | 0 | N | Urinary | N | N |
| 82 | O2/5/16/ 18/20 |  |  |  | N | 2 | Y | GI | N | N |
| 83 | O13/14 |  |  |  | N | 4 | Y | GI | N | N |
| 84 | O10/19 | X |  |  | N | 2 | Y | Urinary | N | N |
| 85 | O6 |  |  |  | N | 2 | Y | GI | N | N |
| 86 | O11/17 |  |  |  | N | 0 | N | Other | N | N |
| 87 | O11/17 | X |  |  | N | NA | Y | GI | N | N |
| 88 | O1 |  |  |  | N | 3 | Y | Urinary | N | Y |
| 89 | O11/17 |  | X |  | N | 8 | Y | GI | N | N |
| 90 | O11/17 | X |  |  | Y | 4 | N | Skin | N | N |
| 91 | O11/17 |  | X |  | N | 2 | N | Urinary | N | N |
| 92 | O7/8 |  | X |  | N | 0 | N | Skin | N | N |
| 93 | NT |  |  |  | N | 4 | Y | GI | N | N |
| 94 | NT |  |  |  | N | 2 | Y | GI | N | N |
| 95 | O3/15 | X |  |  | N | 0 | N | Urinary | N | N |
| 96 | O7/8 |  |  |  | N | 1 | N | Other | N | N |
| 97 | O3/15 |  |  |  | N | 2 | Y | GI | Y |  |
| 98 | O1 |  |  |  | N | 0 | Y | GI | N | N |
| 99 | O6 |  |  |  | N | 2 | Y | GI | N | N |
| 100 | O3/15 | X |  |  | N | 2 | N | Line | N | N |

**cAbs**: Serum contains high titers of anti-O antigen specific IgG2 or IgA. Inhibits: Patient Sera Inhibited HCS- Killing of Cognate Isolate. **CCI:** Charles Comorbidity Index; **Np:** Neutropenia Y: Yes, N: No; GI: Gastrointestinal; AV: aortic valve, VAP: ventilator-associated pneumonia, Line: Central line-associated bloodstream infection.

Supplementary Figure Legends

**Supplementary Figure 1: Antibody response post buffer exchange.** Patient serum was buffer exchanged into an equal volume of 1x PBS. LPS-specific IgG response was measured against the cognate strains serotype of LPS.

**Supplementary Figure 2: LPS extract panel visualized with two methods.** LPS was extracted from nine serotypes of *P. aeruginosa* via the hot phenol method, run on NuPAGE gel and (A) silver stained or (B) western blotting of LPS extracts with a polysera that recognizes all *P. aeruginosa* serotypes other than O10.

**Supplementary Figure 3: HCS killing of isolates during different growth phases.** Twelve isolates, six resistant and six susceptible to HCS killing were grown either to stationary or exponential phase and incubated with either PBS or 50:50 mix of HCS:PBS. Isolates grown to exponential phase tended to be more susceptible to HCS killing, but strains resistant to HCS-killing at stationary phase also were resistant in exponential phase when compared to PBS controls.

**Supplementary Figure 4: O-antigen expression of isolates** (A) LPS was extracted from 34 isolates of *P. aeruginosa*, run on NuPAGE gel and visualized by Western blotting of LPS extracts with O-antigen reactive polysera matched to the strains serotype. Strains with no O-antigen expression marked with an asterix.

**Supplementary Figure 5: Lack of Inhibition of HCS killing.** Serum bactericidal killing of 19 patient’s cognate *P. aeruginosa* strain (PaBi) incubated with either PBS, 50:50 mix of HCS:PBS, 50:50 mix of HCS: matched patient serum (PaBs) or 50:50 patient serum: PBS. Addition of patient serum to HCS did not significantly inhibit HCS-killing. Error bars represent SD.

**Supplementary Figure 6: Inhibition of HCS killing.** Serum bactericidal killing of 14 patient’s cognate *P. aeruginosa* strain (PaBi) incubated with either PBS, 50:50 mix of HCS:PBS, 50:50 mix of HCS: matched patient serum (PaBs) or 50:50 patient serum: PBS. Addition of patient serum to HCS inhibited HCS -killing significantly based on AUC calculations. n = 3 for all sera. *, P < 0.05; **, P < 0.01; ***, P < 0.001; ****, P < 0.0001. Error bars represent SD.

Supp Fig. 1


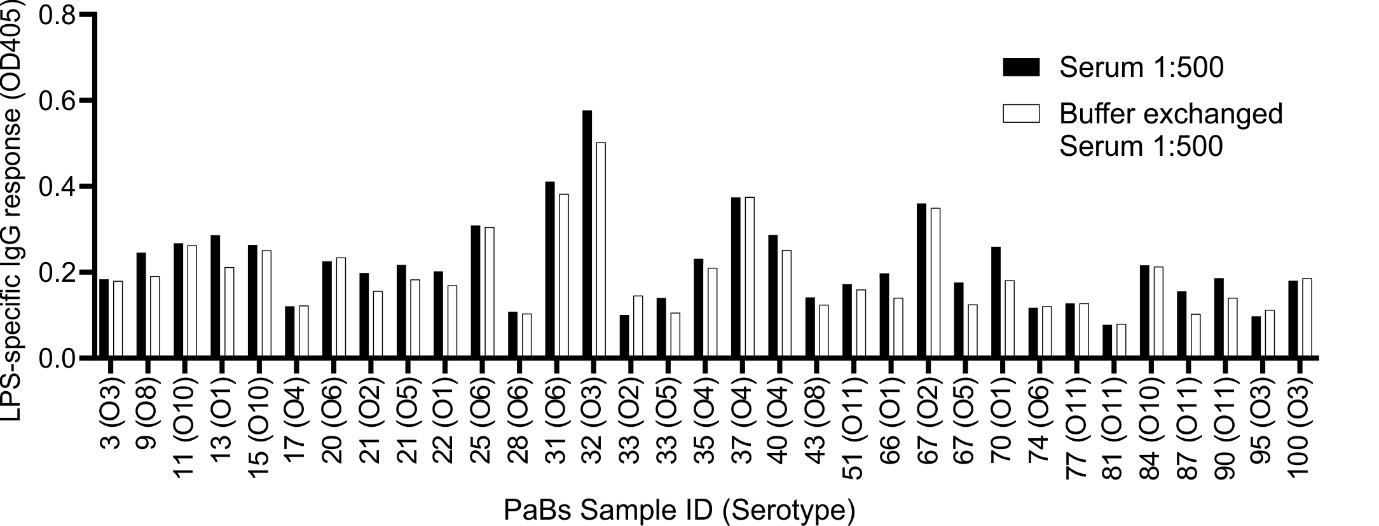


Supp Fig. 2


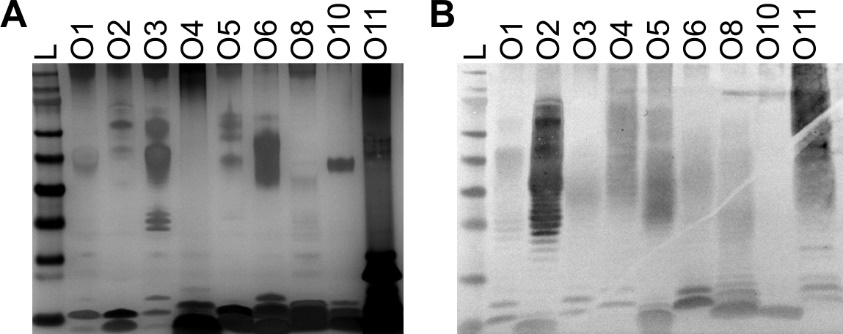


Supp Fig.3


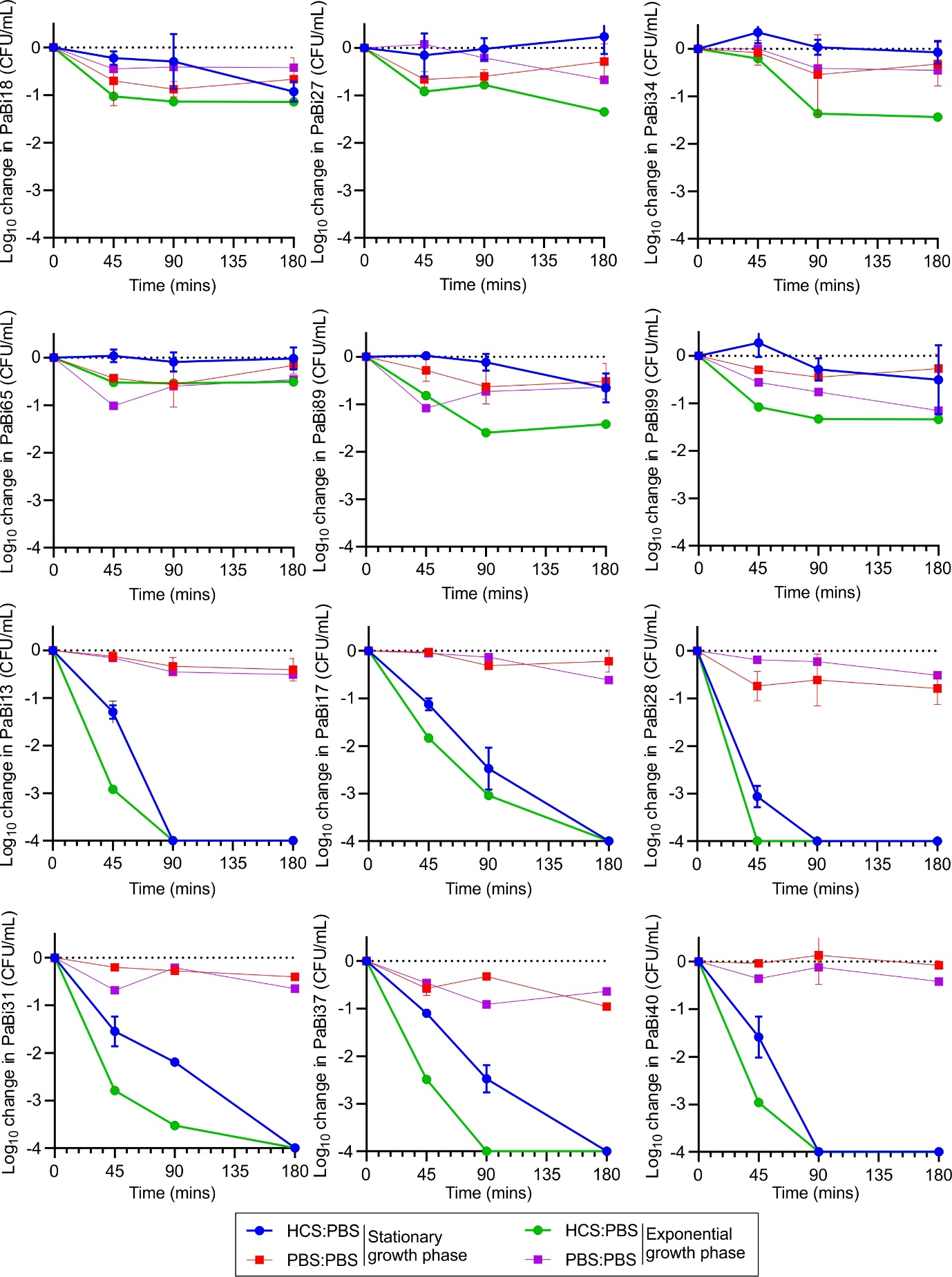


Supp Fig. 4


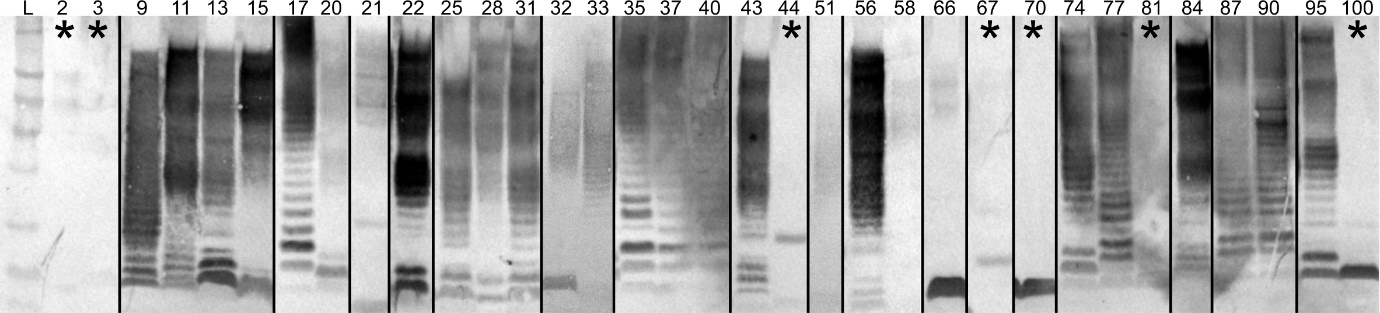


Supp Fig 5


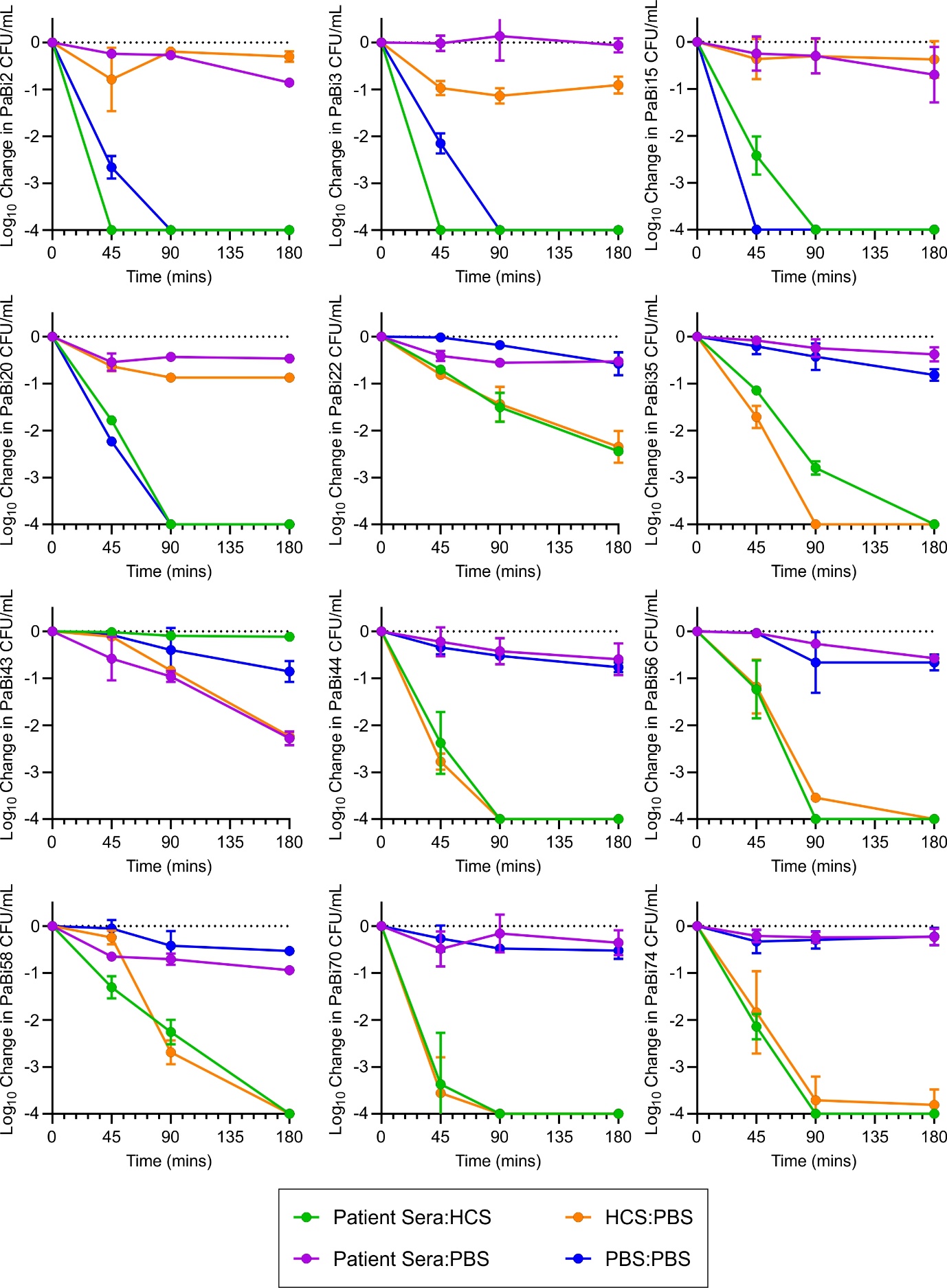


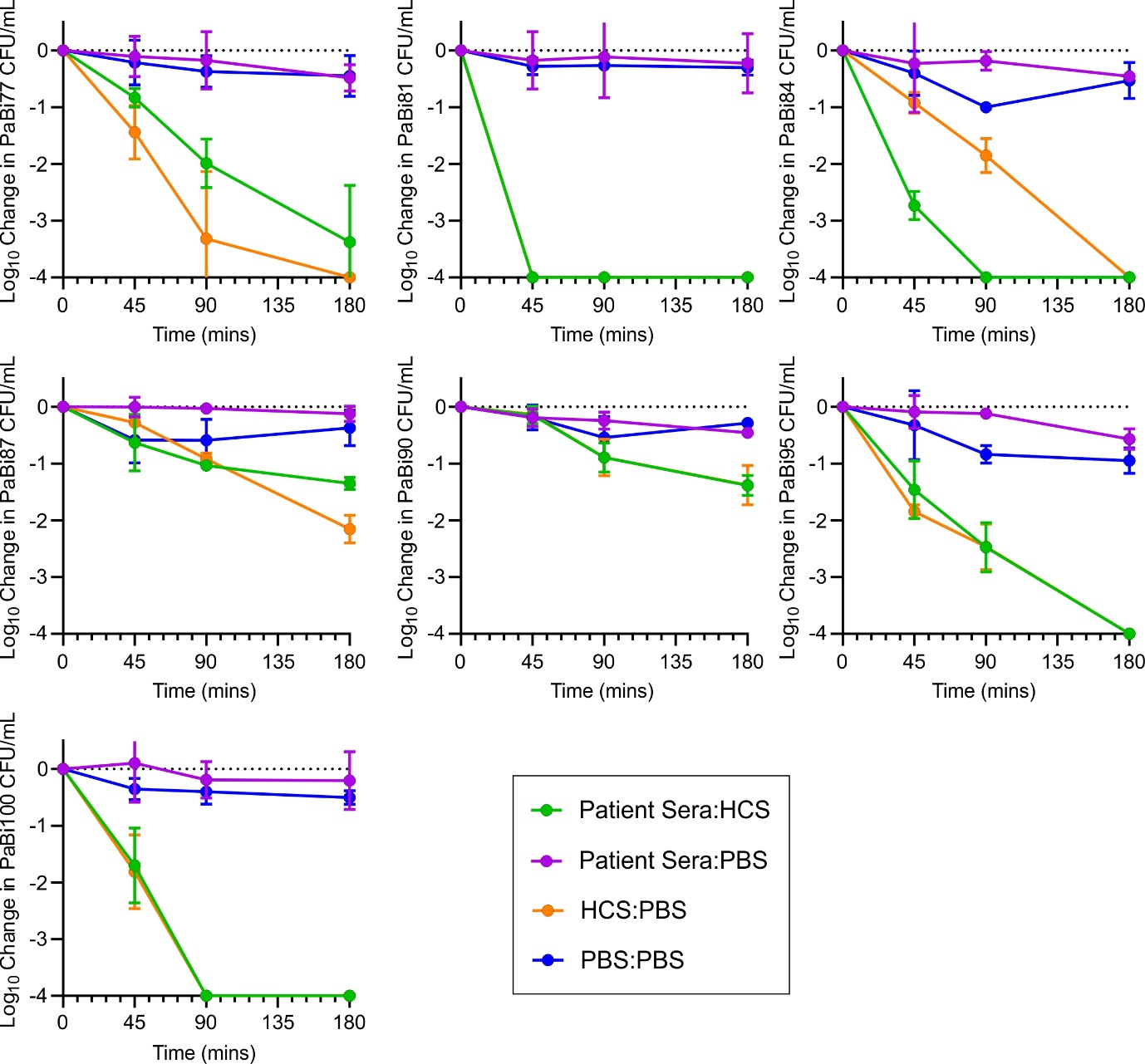


Supp. Fig. 6


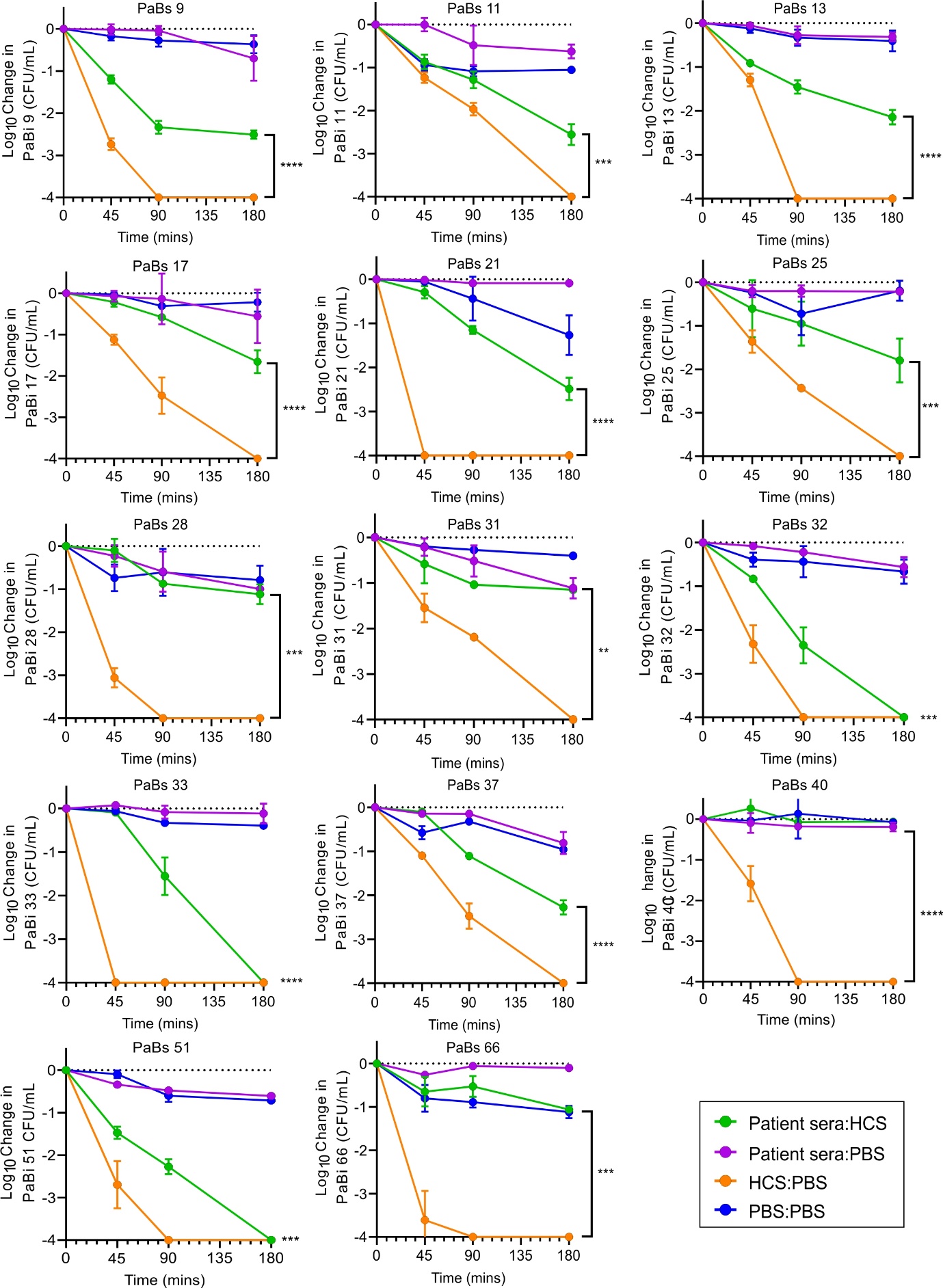

Supplement: jiad457_Supplementary_Data [file jiad457_supplementary_data.docx]
